# Efficacy and Safety of *Lactobacillus Plantarum* C29-Fermented Soybean (DW2009) in Individuals with Mild Cognitive Impairment: A 12-Week, Multi-Center, Randomized, Double-Blind, Placebo-Controlled Clinical Trial

**DOI:** 10.3390/nu11020305

**Published:** 2019-02-01

**Authors:** Yun-Ha Hwang, Shinwon Park, Jong-Woo Paik, Soo-Wan Chae, Dong-Hyun Kim, Doc-Gyun Jeong, Eunji Ha, Myeongju Kim, Gahae Hong, Soo-Hyun Park, Su-Jin Jung, Sang-Min Lee, Kyu-Heum Na, Jungyoon Kim, Young-Chul Chung

**Affiliations:** 1 DONGWHA Pharm Research Institute, 35-71, Topsil-ro, Giheung-gu, Yongin-si 17084, Korea; yunha.hwang@dong-wha.co.kr (Y.-H.H.); docgyun.jeong@dong-wha.co.kr (D.-G.J.); kyuheum.na@dong-wha.co.kr (K.-H.N.); 2 Ewha Brain Institute and Department of Brain & Cognitive Sciences, Ewha Womans University, 52, Ewhayeodae-gil, Seodaemun-gu, Seoul 03760, Korea; shinwon.s.park@gmail.com (S.P.); eunji.i.ha@gmail.com (E.H.); myeongju.j.kim@gmail.com (M.K.); gahae.h.hong@ewha.ac.kr (G.H.); 3 Department of Psychiatry, College of Medicine, Kyung Hee University, 26, Kyungheedae-ro, Dongdaemun-gu, Seoul 02447, Korea; paikjw@khu.ac.kr (J.-W.P.); maumdoctor@gmail.com (S.-M.L.); 4 Clinical Trial Center for Functional Foods, Chonbuk National University Hospital, 20, Geonjiro Deokjin-gu, Jeonju-si 54907, Korea; swchae@jbctc.org (S.-W.C.); shpark@jbctc.org (S.-H.P.); sjjeong@jbctc.org (S.-J.J.); 5 Department of Life and Nanopharmaceutical Sciences, College of Pharmacy, Kyung Hee University, 26, Kyungheedae-ro, Dongdaemun-gu, Seoul 02447, Korea; dhkim@khu.ac.kr; 6 Research Institute of Clinical Medicine of Chonbuk National University-Biomedical Research Institute of Chonbuk National University Hospital, 20, Geonjiro Deokjin-gu, Jeonju-si 54907, Korea

**Keywords:** Mild cognitive impairment, *Lactobacillus plantarum* C29-fermented soybean (DW2009), Cognitive impairment, Brain-derived neurotrophic factor

## Abstract

Early intervention using dietary supplements may be effective in alleviating cognitive impairment among individuals with mild cognitive impairment (MCI). This study investigated the efficacy and safety of *Lactobacillus plantarum* C29-fermented soybean (DW2009) as a nutritional supplement for cognitive enhancement. One hundred individuals with MCI were randomly assigned to take DW2009 (800 mg/day, *n* = 50) or placebo (800 mg/day, *n* = 50) for 12 weeks. The primary outcome measure was change in the composite score of cognitive functions related to memory and attention, measured by computerized neurocognitive function tests. Associations between changes in serum brain-derived neurotrophic factor (BDNF) levels and cognitive performance for each treatment group were evaluated. Compared to the placebo group, the DW2009 group showed greater improvements in the combined cognitive functions (*z* = 2.36, *p* for interaction = 0.02), especially in the attention domain (*z* = 2.34, *p* for interaction = 0.02). Cognitive improvement was associated with increased serum BDNF levels after consumption of DW2009 (*t* = 2.83, *p* = 0.007). The results of this clinical trial suggest that DW2009 can be safely administered to enhance cognitive function in individuals with MCI. Increased serum BDNF levels after administering DW2009 may provide preliminary insight into the underlying effects of cognitive improvement, which suggests the importance of the gut-brain axis in ameliorating cognitive deficits in MCI.

## 1. Introduction

Mild cognitive impairment (MCI) refers to a state of cognitive deterioration that precedes the clinical diagnosis of Alzheimer’s disease (AD) and other dementias, which does not yet compromise daily functioning [1]. The estimated prevalence of MCI among people older than 65 years of age ranges from 3% to 20% in population-based epidemiological studies [2]. Previous studies have shown that people with MCI are at an increased risk for developing dementia [3,4], thus early awareness of MCI may facilitate symptomatic alleviation and slow the disease progression. Previous research has shown that probiotic supplementation has beneficial effects on cognitive function in patients with AD [5], which may be developed as a dietary supplement for MCI. Dietary habits and regular consumption of functional foods before the onset of AD may provide health benefits and reduce disease risk.

*Lactobacillus plantarum* C29 is an anti-inflammatory probiotic isolated from kimchi, a traditional Korean side dish made from fermented vegetables. *Lactobacillus plantarum* C29 effectively increased cognitive performance in aged rats and ameliorated scopolamine-induced memory impairment in mice by inhibiting brain inflammation due to excessive lipopolysaccharide in the gut microbiota and restoring hippocampal brain-derived neurotrophic factor (BDNF) expressions [6,7]. In addition, the cognitive enhancement effects of *Lactobacillus plantarum* C29 were demonstrated in mice with 2,4,6-trinitrobenzenesulfonic acid (TNBS)-induced memory impairment [8].

As its effects have been reported to contribute to the healthy microbiota through antibacterial and probiotic properties, *Lactobacillus plantarum* C29 was used to ferment defatted soybean to produce DW2009. Consumption of soybeans was reported to enhance memory in humans [9] and the bioactive ingredients contained in soybean, isoflavones and saponins, also exhibited memory-enhancing effects [10,11]. Although soybean itself is nutritional, undergoing the process of fermentation with lactic acid bacteria is expected to augment metabolic reactions resulting in increased isoflavone absorption and antioxidant capacity [12]. Recently, we confirmed the positive effects of DW2009 on cognition in a preclinical study, where oral administration of DW2009 not only normalized the disturbed gut microbiota composition but also increased hippocampal BDNF levels in AD mouse models [13]. Although both *Lactobacillus plantarum* C29 and DW2009 increased cognitive function, DW2009 more effectively suppressed the accumulation of amyloid-β plaques and alleviated memory impairment in AD mice [13]. However, the cognitive enhancement effects of DW2009 in MCI have not yet been widely studied in the human population.

In the present study, we conducted a 12-week, double-blind, placebo-controlled, randomized clinical trial (RCT) to assess the efficacy and safety of DW2009 as a nutritional supplement for cognitive enhancement in individuals with MCI. Since memory deficits are the core symptoms in MCI [14,15], and attention is the basis for encoding of memory, we examined the effects of DW2009 on attention and memory functions. The primary outcome measure was change in the composite score of cognitive functions including attention, working memory, and verbal memory. Serum BDNF levels were evaluated in association with change in cognitive function.

## 2. Materials and Methods

### 2.1. Participants

Physically healthy men and women diagnosed with MCI according to the Diagnostic and Statistical Manual of Mental Disorders, 5th edition (DSM-5), were recruited at Chonbuk National University Hospital and Kyung Hee University Hospital. A total of 133 potential participants were screened for eligibility. Inclusion criteria were as follows: (1) a subjective complaint of memory impairment; (2) age between 55 and 85 years; (3) memory index scores that fell greater than 1 standard deviation (SD) below the mean in age- and education-matched controls, assessed with the Korean version of the Consortium to Establish a Registry for Alzheimer’s Disease Assessment Packet (CERAD-K). Participants were screened out when they met any of the following exclusion criteria: (1) presence of any uncontrolled, clinically significant medical or psychiatric disease; (2) diagnosis of diseases accompanied by cognitive dysfunction such as dementia, Parkinson’s disease, or stroke; (3) use of medications which could affect cognitive functions, such as antipsychotics, nootropics, or tricyclic antidepressants, within 4 weeks prior to the screening of the study; and (4) use of nutritional supplements to enhance cognitive function within 2 weeks prior to the screening of the study. The study protocol and consent forms were approved by the Institutional Review Boards of Chonbuk National University Hospital and Kyung Hee University Hospital, and all participants provided written informed consent to participate in the study. This study is registered in the Clinical Research Information Service of South Korea (board approval number: KCT0002346).

### 2.2. Procedure

**Figure 1:**
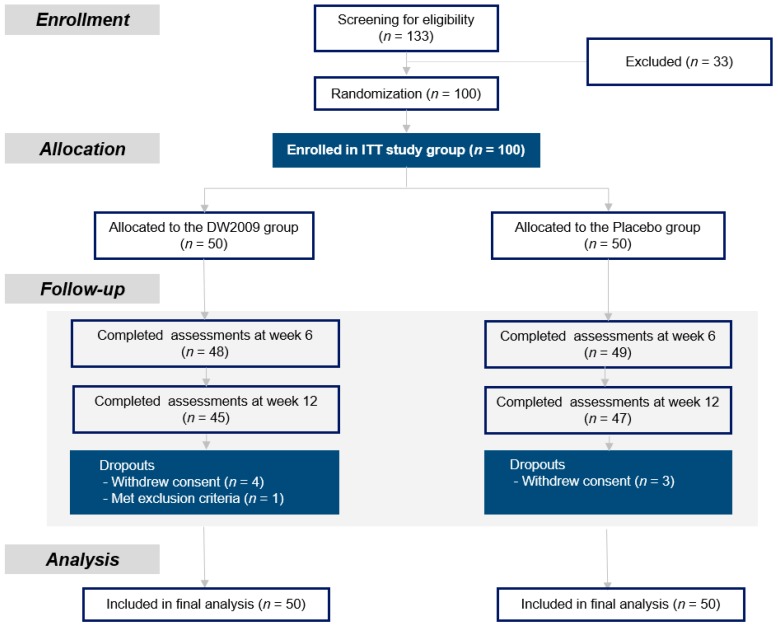
Screening, randomization, and allocation of participants to the DW2009 or placebo groups.

After screening for eligibility, 100 participants were enrolled in the 12-week double-blind, placebo-controlled RCT. Participants were randomly assigned to DW2009 (800 mg/day, *n* = 50) or Placebo (*n* = 50) groups (Figure 1). Random allocation sequence was generated by using the PROC PLAN procedure of the SAS version 9.4 program (SAS Institute, Cary, NC, USA) and was also supervised by the Korean National Institute of Health. Allocation concealment and double-blinding were carried out in accordance with the International Conference on Harmonization Good Clinical Practice (ICH GCP) guideline.

The placebo capsules were made of cellulose and manufactured to be indistinguishable from the treatment capsule in appearance. After receiving the treatment or placebo capsules at the baseline visit, follow-up visits were scheduled at 6 and 12 weeks. During the study period, participants were instructed to orally intake 2 capsules, once a day, to refrain from taking nutritional supplements for cognitive enhancement, and to report if they began any new treatment. If the treatment included intake of antipsychotics, antidepressants, estrogen replacement therapy, or neurodegenerative disease drugs, then study participation was discontinued.

Based on the results from our previous animal study that showed cognitive enhancement effects of DW2009 and considering the acceptable daily intake of *Lactobacillus* genus for humans without appreciable adverse effects [16], the dose of 800 mg/day was selected. DW2009 is a mixture of fermented soybean powder and *Lactobacillus plantarum* C29 freeze-dried powder. Fermented soybean powder was prepared as below: defatted soybean (6%) was inoculated with *Lactobacillus plantarum* C29, fermented at 37 °C for 24 h, and then freeze-dried. *Lactobacillus plantarum* C29 freeze-dried powder was obtained from the culture in MRS (De Man, Rogosa, Sharpe agar) broth. This mixture contains 62.5% fermented soybean powder and 1.25 × 10^10^ CFU/g or more of *Lactobacillus plantarum* C29. Quality control including shelf-life testing during the entire study period was guaranteed through several validated analytical methods.

### 2.3. Outcome Measures

#### 2.3.1. Measurement of Cognitive Functions

The computerized neurocognitive function tests (CNT) were performed at baseline and repeated at 12 weeks, the end of the study period. The primary outcome measure of this study was change in the composite score of cognitive functions including attention, working memory, and verbal memory. Cognitive domains of verbal memory function, attention, and working memory function were evaluated by the verbal learning test (VLT), auditory continuous performance test (ACPT), and digit span test (DST), respectively. These tests were part of a validated computerized test battery consisting of 18 tests [17]. As memory deficits are core symptoms of MCI and attention is a prerequisite for memory encoding, decline in these cognitive functions are commonly reported in MCI [14,15]. Therefore, we chose the most appropriate subtests to examine these cognitive domains of participants with MCI.

For the VLT, 15 words are read aloud by an examiner and the participants are required to memorize the list [18]. Participants receive scores for immediate recall, delayed recall, and recognition to evaluate verbal memory function [18]. The ACPT measures selective and sustained attention by asking participants to respond by pressing a button as soon as they hear the target stimulus [19]. Correct and incorrect detection, omission errors, and reaction time are measured while participants listen to random numbers for 9 min and respond to the target stimulus [19]. The DST was used to evaluate working memory by asking participants to immediately recall a sequence of numbers [18]. Outcome measures of list memory and tracking scores indicate the number of correct responses and the longest digit span length correctly recalled, respectively [18].

Composite *z* scores for each cognitive domain were calculated using the group mean scores and SD of the baseline placebo group. To calculate the *z* scores, the difference between each individual raw score and mean score was divided by the SD. If necessary, test scores were reversed so that positive z scores indicate better performance. Composite *z* scores for the verbal memory function were calculated by averaging *z* scores of immediate recall, delayed recall, and recognition of the VLT. Measurements of correct detection, incorrect detection, omission error, and reaction time of the ACPT were standardized to *z* scores and averaged to make composite *z* scores for the attention function. The standardized *z* scores of list memory and tracking scores of the DST were averaged to calculate composite *z* scores for the working memory function. Finally, as a primary outcome measure, a total composite score of the measured cognitive functions was calculated by averaging the *z* scores of the three cognitive domains.

#### 2.3.2. Safety Measures

Safety and tolerability of study supplements were monitored and documented at every visit. Any untoward medical occurrence during the study was recorded as an adverse event regardless of association with the clinical trial product DW2009 or placebo. We recorded any symptoms reported by the participants, along with vital signs and laboratory tests. Using a non-directive questioning approach, we assessed the participants’ spontaneous and self-reported adverse events and specific inquiries regarding health problems. Severity of the reported symptoms were evaluated as mild, moderate, and severe. Symptoms were considered as mild if treatment was not required and activities of daily life were not impaired. Moderate symptoms caused impairment in daily life, which were expected to fully recover with treatment. Severe symptoms indicate serious side effects that required immediate treatment and may cause long-term side effects. Height, weight, and vital signs, including blood pressure and pulse rate, were measured at every visit. Comprehensive physical and neurological examinations, electrocardiograms and routine laboratory examinations, such as the complete blood cell count and blood chemistry tests, including but not limited to aminotransferases, blood urea nitrogen, creatinine, total cholesterol, glucose, and albumin levels, were conducted at baseline and at the final visit.

#### 2.3.3. Measurement of Serum BDNF levels

Blood samples for serum BDNF analysis were collected in serum-separating tubes at visit 2 and visit 4. After being centrifuged at 1000 × g for 15 min, the separated supernatant was stored at −80 °C until assay. Serum samples were sent to the Green Cross Reference Laboratory within 1 month for analysis. Serum BDNF level was assayed using a Quantikine enzyme-linked immunosorbent assay (ELISA) kit (R&D Systems, Minneapolis, MN, USA), according to the manufacturer’s instructions. A microplate reader (Molecular Device), set at 450 nm, was used to determine serum BDNF values. The sensitivity of the assay was 20 pg/mL. The intra-assay and inter-assay variations (CV) were 5% and 9%, respectively.

#### 2.3.4. Fecal Microbiota Analysis

Fecal samples were randomly collected from 92 participants (45 persons in DW2009-treated group and 47 persons in placebo group), at the baseline and follow-up visits to analyze changes in fecal microbiota. Gut microbiota DNA was isolated from these collected feces, using a Qiagen DNA stool mini kit (Qiagen, Hilden, Germany). The number of samples applicable for qPCR was 38–40 in the placebo group and 37–38 in the DW2009-treated group. qPCR was performed with 20 ng of total DNA isolated from the feces with the Takara thermal cycler, using SYBR premix agents (Takara, Shiga, Japan). Thermal cycling conditions were as follows: activation of DNA polymerase at 95 °C for 5 min, followed by 40 cycles of denaturation at 95 °C for 15 s, primer annealing at 60 °C for 60 s, and extension at 72 °C for 30 s. The normalized expression of the assayed genes, with respect to bacterial rRNA, was computed for all samples using the Microsoft Excel data spreadsheet. Primers were used as follows: *Bifidobacterium* spp. forward 5′-CGCGTCYGGTGTGAAAG-3′ and reverse 5’- CCCCACATCCAGCATCCA-3′; *Lactobacillus* spp. forward 5′-GAGGCAGCAGTAGGGAATCTTC-3′, reverse 5′-GGCCAGTTACTACCTCTATCCTTCTTC-3′; *Clostridium* spp. forward 5′-ACGCTACTTGAGGAGGA-3′ and reverse 5′-GAGCCGTAGCCTTTCACT-3′; and bacterial 16S rRNA forward 5′-TCGTCGGCAGCGTCAGATGTGTATAAGAGACAGGTGCCAGCMGCCGCGGTAA-3′, reverse 5′-GTCTCGTGGGCTCGGAGATGTGTATAAGAGACAGGGACTACHVGGGTWTCTAAT-3′.

### 2.4. Statistical Analysis

All data analysis was performed on an intent-to-treat basis. Baseline differences in demographic and clinical characteristics between the groups were examined using independent t tests, χ^2^ tests, or Fisher’s exact tests. Analyses for the primary efficacy measure were performed using a mixed-effects model repeated-measures analysis. Treatment group (DW2009, placebo), visit (baseline, follow-up), and visit-by-group interaction were included as fixed effects, and age, sex and baseline scores were included as covariates. The within-subject factor was treated as a random effect. Analysis of the full, incomplete data set with maximum likelihood estimation was performed to handle missing values. Associations between changes in BDNF levels in serum and cognitive performance were assessed for each treatment group using robust regression. Significant changes in fecal bacteria population were examined using one-way ANOVA. The analysis of safety data was conducted on all participants who took at least one dose of study supplement.

## 3. Results

### 3.1. Participant Characteristics

Among the 100 participants, a total of 92 patients completed the study. The dropout rates did not significantly differ between the DW2009 group and placebo group (10.0% vs. 6.0%, respectively). Baseline demographic and clinical characteristics were similar in both groups, as shown in Table 1.

### 3.2. Adherence to Treatment

Compliance rate was measured by counting the returned clinical trial products and calculating the percentage of consumed products to the prescribed dosage. It was calculated only for participants who completed the study. Both DW2009 and placebo groups showed a compliance rate of 91.1% and 93.1%, respectively. There was no significant difference of compliance rate between the two groups (*p* = 0.43).

### 3.3. Primary Outcome Measure

**Figure 2:**
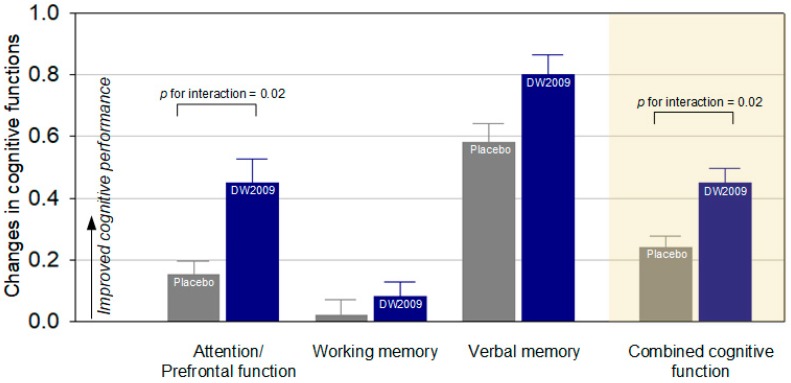
Differences in changes in composite *z* scores of each cognitive domain and combined cognitive function between the placebo-treated and DW2009-treated groups. Error bars indicate standard errors.

Compared to individuals in the placebo group, those who received DW2009 showed greater cognitive improvement at the end of the study. The improvement rate in the combined cognitive function was greater in individuals in DW2009 than that of the placebo group (*z* = 2.36, *p* for interaction = 0.02). Among these domain composite scores, change in the attention composite score was statistically significant (*z* = 2.34, *p* for interaction = 0.02). The scores of each individual measure comprising each domain and composite scores of the attention, working memory, verbal memory and combined cognitive function are presented in Table 2 and Figure 2.

### 3.4. Associations between Changes in Serum BDNF Levels and Cognitive Performance

**Figure 3:**
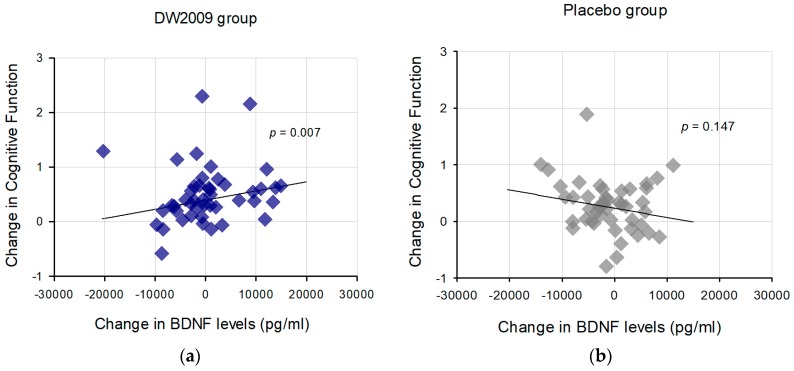
Association between change in brain-derived neurotrophic factor (BDNF) levels and combined cognitive function in (**a**) DW2009 treatment group and (**b**) placebo group. Composite z scores for the combined cognitive function were calculated by averaging z scores of the measured cognitive domains (attention, working memory, and verbal memory).

For the DW2009 group, change in serum BDNF levels were positively associated with change in the combined cognitive function (*t* = 2.83, *p* = 0.007), after controlling for age, sex, education level and baseline cognitive performance. There was no significant association between change in serum BDNF levels and cognitive performance in the placebo group (*t* = −1.48, *p* = 0.15) (Figure 3). The mean change in serum BDNF levels for DW2009 group was 412.7 pg/mL (SD = 7212.4), while for the placebo group it was −1034.3 pg/mL (SD = 5644.5).

### 3.5. Change in Gut Microbiota Composition

In regards with the gut microbiota composition, the lactobacilli population was significantly increased in the DW2009 group (*p* = 0.045), but not in the Placebo group (*p* = 0.334). There were no significant changes in the bifidobacteria (DW2009: *p* = 0.789; Placebo: *p* = 0.160) and clostridia populations (DW2009: *p* = 0.936; Placebo: *p* = 0.259), for both DW2009 and Placebo groups (Table 3).

### 3.6. Safety and Tolerability

The overall frequency of all reported adverse events during the study participation were seven cases in the DW2009 group and five cases in the placebo group. One of the adverse events reported in the DW2009 group was classified as a serious adverse event (lung cancer) and the participant withdrew his participation to receive treatment. Other adverse events observed in the DW2009 group were dizziness, stomach aches, headaches, gastritis, erectile dysfunction and seborrheic dermatitis, all of which were classified as a mild adverse event, except for dizziness. Dizziness was classified as a moderate adverse event possibly related to clinical trial product intake, and the participant experiencing dizziness withdrew from participation and recovered. For gastritis and seborrheic dermatitis, co-administration of medication was allowed during study period. Adverse events observed in the placebo group were irregular bowel movement, stomach aches, and erectile dysfunction, all of which were classified as a mild adverse event. The rest of the adverse events were spontaneously resolved without specific interventions during study participation. There were no differences in vital signs, body mass index and laboratory findings between the treatment groups (Table 4 and Table 5).

## 4. Discussion

This double-blind, placebo-controlled RCT examined the effects of DW2009 as a dietary supplement for individuals with MCI. Oral administration of DW2009 was found to improve cognitive performance, especially that of attention. In addition, there were significant associations between cognitive enhancement and change in serum BDNF levels in the DW2009 group. To the best of our knowledge, this is the first study to investigate the cognitive enhancement effects of DW2009 in humans and our findings extend the previously reported evidence of cognitive improvement from animal studies.

Defatted soybean was fermented with *Lactobacillus plantarum* C29, a gram-positive and acid-tolerant lactic acid bacterium, commonly found in fermented food and in the gastrointestinal microbiota of living species. Our study results found an improvement in the composite score of attention and working/verbal memory after consumption of DW2009. Attention and memory are commonly reported to disproportionately deteriorate in MCI and AD [15]. There was a significant increase in the attention function among those who consumed DW2009. Regarding the memory function, there was a meaningful trend towards improvement, despite the relatively short period of 12 weeks to test for effects in patients who were only mildly impaired. The beneficial effects of DW2009 on cognitive performance in individuals with MCI are in line with our previous preclinical study conducted in mice. Oral administration of DW2009 in the AD mouse model attenuated memory impairment and inhibited amyloid-β expression, a major pathologic finding of AD, by regulating gut microbiota composition and increasing BDNF expression [13]. Increased levels of serum BDNF expression among those who took DW2009 were also reported in the current study, which may suggest a potential change in brain-tissue BDNF levels that may have mediated cognitive enhancement, as in the previous AD mouse model.

BDNF is known to modulate synaptic transmission and neuronal plasticity [20,21,22] and protect against neuro-inflammation and neuronal apoptosis [23]. As such, higher BDNF levels are robustly linked with better memory [24] and executive function [25], whereas decreased levels of BDNF have been associated with AD and MCI [26,27]. A similar pattern was observed in the current study in which increased BDNF levels were associated with improvement in the combined cognitive function. Interestingly, this effect was only found for the group that consumed DW2009, which suggests that DW2009 may improve cognitive function by increasing BDNF expression. This speculation is supported by a previous study reporting an increase in TNBS-suppressed expression of BDNF levels and inhibition of TNBS-induced NF-κB activation in the hippocampus, along with memory improvement in mice treated with *Lactobacillus plantarum* C29 [8].

Involvement of short-chain fatty acids, G protein coupled receptors (GPCRs), and neurotransmitters are proposed methods by which the gut microbiota influence BDNF levels. The gut microbiota produces metabolites, such as short-chain fatty acids, that may influence various brain regions by activation of GPCRs [28]. In addition, the gut microbiota can generate neurotransmitters or their derivatives, such as gamma-aminobutyric acid (GABA) and butyric acid [29,30], which have been shown to influence BDNF expression. As bacteria can generate various neurotransmitters, this may be a possible underlying mechanism by which the gut microbiota influences BDNF expression [28]. Previous research has shown that *Lactobacillus plantarum* C29 induced BDNF expression via proliferation of lactobacilli and bifidobacteria in gut microbiota composition in vivo [8]. *Lactobacillus plantarum* C29 directly stimulated BDNF expression in SH-Sy4H cells in vitro [31]. Based on these findings, *Lactobacillus plantarum* C29 may induce BDNF expression by indirectly regulating gut microbiota or directly biosynthesizing neurotransmitters or its derivatives.

Gut microbiota composition fluctuations can be influenced by internal and external factors of the host, such as diet, drug use, stress, and aging [32,33]. Despite the possible large variance in gut microbiota composition between individuals, DW2009 consumption significantly increased the number of lactobacilli in the gut bacterial composition. Assay of fecal bacteria suggests that oral administration of DW2009 may affect the gut microbiota composition by stimulating the proliferation of the gut lactobacilli population. These results suggest that *Lactobacillus plantarum* C29, which was contained in DW2009, can be resistant to stomach acid, bile, digestive enzymes, and the gut microbiota barrier. As most of the lactobacillus species have a “generally regarded as safe” (GRAS) status [34], they have been frequently used as probiotics. Thus, an increase in beneficial gut bacteria, such as *Lactobacillus* spp. in the gastrointestinal (GI) tract, may have alleviated cognitive decline. The microbiota-gut-brain axis has been reported to mediate changes in behavior, including cognitive decline [35], as well as regulate neuropsychiatric diseases [36]. Our study results are in line with previous studies that suggest potential effects of interactions between the gut microbiota and brain on human behavior and cognition.

To the best of our knowledge, this is the first double-blind, placebo-controlled RCT to examine the efficacy of DW2009 on cognitive performance in individuals with MCI. However, there are several limitations that should be addressed. First, not all of the critical factors related to MCI were evaluated in the study. While participants were tested in the cognitive domains of attention, working memory and verbal memory, other cognitive domains were not assessed partly due to limited time and resources available during the participants’ visits to the clinical trial center. The lack of neuro-radiological or genetic data precludes deeper investigation into putative biomarkers of amyloid beta deposition or the previously reported APOE4-related risk of MCI and dementia. Second, although improvement in the composite score of cognitive functions was found during the 12-week intervention period, the results should be cautiously interpreted. Additional RCTs for a longer period of administration is needed to ensure a more long-term effect of DW2009 on cognitive enhancement. However, many previous studies have reported cognitive improvements after dietary supplementation of 12 weeks or less in MCI [37,38,39], indicating that the 12-week intervention paradigm is widely implemented to test for cognitive enhancement effects of dietary supplementation. Third, although correlations between change in serum-derived BDNF levels and cognitive performance may provide preliminary evidence on the brain-based mechanism of the effects of DW2009 on cognition, further longitudinal or animal experimental studies are required to establish causation. Moreover, future studies may implement multiple time points and an extended intervention period to assess the effects of DW2009 consumption on MCI. Finally, although we demonstrated an effect with a fixed dose of 800 mg per day, there may be potential dose-dependent responses, which can be examined in future studies.

In conclusion, the results of this study demonstrated the efficacy and safety of DW2009 supplementation in improving cognitive function in individuals with MCI. As its cognitive enhancement effects have been confirmed in both humans and mice, it can be considered a promising option to ameliorate cognitive deficits in MCI.

## Figures and Tables

**Table 1:** Baseline demographic and clinical characteristics of study participants (*n* = 100).

Characteristics	Treatment Groups		*p*-Value
	Placebo (*n* = 50)	DW2009 Group (*n* = 50)	
Demographic characteristics			
Age, mean, (SD)	69.2 (7.00)	68.0 (5.12)	0.35
Women, No. (%)	36 (72.0)	30 (60.0)	0.21
Education, No. (%)			
0–11 years	25 (50.0)	30 (60.0)	0.43
12 years	17 (34.0)	16 (32.0)	
13 years or more	8 (16.0)	4 (8.00)	
Marital status, No. (%)			
Married	50 (100)	49 (98.0)	1.00
Divorced, widowed, or separated	0 (0.00)	1 (2.00)	
Clinical characteristics			
Auditory continuous performance test ^1^			
Reaction time	0.77 (0.08)	0.79 (0.08)	0.13
Correct response	127 (9.41)	121 (20.2)	0.04
Incorrect response	6.29 (6.15)	9.22 (8.12)	0.05
Omission error	7.71 (9.40)	14.2 (20.2)	0.04
Digit span test			
List memory score	7.44 (3.35)	7.34 (3.66)	0.89
Tracking score	5.26 (1.23)	5.34 (1.45)	0.77
Verbal learning test			
Immediate recall	9.04 (2.22)	8.30 (2.35)	0.11
Delayed recall	7.30 (2.62)	6.84 (2.91)	0.41
Recognition	11.3 (2.34)	10.6 (3.21)	0.19

^1^ Data from one subject of the placebo-administered group was not available. *p*-values were calculated using the independent *t*-test for continuous variables and chi-squared test or Fisher’s exact test for categorical variables. SD: standard deviation; No.: number.

**Table 2:** Composite scores of attention, working memory, and verbal memory function measured using the computerized neurocognitive function tests between the groups at baseline and after 12 weeks of treatment.

	Placebo		DW2009		*z*	*p* for Interaction	*p* for Group	*p* for Time
	Baseline (*n* = 50)	Follow-up (*n* = 47)	Baseline (*n* = 50)	Follow-up (*n* = 45)				
Attention/Prefrontal function ^1^								
Reaction time	0.77 (0.08)	0.78 (0.08)	0.79 (0.08)	0.77 (0.09)	−2.99	0.003	0.50	0.25
Correct response	127 (9.41)	130 (8.38)	121 (20.2)	125 (16.2)	1.59	0.11	0.38	0.06
Incorrect response	6.29 (6.15)	4.96 (5.27)	9.22 (8.12)	6.00 (5.84)	−1.64	0.10	0.32	0.07
Omission error	7.71 (9.40)	5.22 (8.38)	14.2 (20.2)	9.56 (16.2)	−1.62	0.11	0.38	0.06
Domain composite score	0.00 (0.82)	0.15 (0.70)	−0.54 (1.44)	−0.09 (1.05)	2.34	0.02	0.24	0.11
Working memory function								
List memory score	7.44 (3.35)	7.68 (3.65)	7.34 (3.66)	7.87 (3.57)	0.92	0.36	0.81	0.29
Tracking score	5.26 (1.23)	5.21 (1.30)	5.34 (1.45)	5.38 (1.39)	0.79	0.43	0.82	0.87
Domain composite score	0.00 (0.98)	0.02 (1.06)	0.02 (1.12)	0.11 (1.08)	0.63	0.53	0.90	0.69
Verbal memory function								
Immediate recall	9.04 (2.22)	10.4 (2.48)	8.30 (2.35)	10.2 (2.77)	1.09	0.28	0.67	<0.001
Delayed recall	7.30 (2.62)	9.19 (3.10)	6.84 (2.91)	9.11 (2.81)	0.53	0.60	0.72	<0.001
Recognition	11.3 (2.34)	12.4 (1.94)	10.6 (3.21)	12.2 (2.18)	1.16	0.25	0.53	<0.001
Domain composite score	0.00 (0.87)	0.58 (0.93)	−0.28 (0.99)	0.52 (0.98)	1.26	0.21	0.66	<0.001
Combined cognitive function								
Domain composite score	0.01 (0.68)	0.25 (0.65)	−0.27 (0.95)	0.18 (0.79)	2.36	0.02	0.55	<0.001

^1^ Data from one subject of the placebo-administered group was not available. For domain composite scores, data are presented in (*z* score (standard deviations, SD)). If necessary, test scores were reversed to indicate better performance with positive z scores. For individual measures comprising each domain, data are presented in (mean (SD)). Composite z scores for the attention function were calculated by averaging z scores of correct detection, incorrect detection, omission error, and reaction time of the auditory continuous performance test. Composite z scores for the working memory function were calculated by averaging z scores of the list memory scores and tracking scores of the digit span test. Composite z scores for the verbal memory function were calculated by averaging z scores of immediate recall, delayed recall, and recognition of the verbal learning test. Composite z score for the combined cognitive function was calculated by averaging z scores of all cognitive domains (attention, working memory, and verbal memory). Analyses for primary efficacy measures of each cognitive domain were performed using a mixed-effects model repeated-measures analysis. Treatment group, visit, and treatment group-by-visit interaction were included as fixed effects, while the within-subjects factor was included as a random effect. Age, sex, educational level, and baseline composite scores were included as covariates.

**Table 3:** Effect of DW2009 on the bifidobacteria, lactobacilli, and clostridia.

Gut Microbiota Composition	Placebo Group			DW2009 Group		
	Baseline	Follow-up	*n*	Baseline	Follow-up	*n*
*Bifidobacterium* spp.	2.99 × 10^11^ (1.13 × 10^12^)	4.46 × 10^10^ (1.11 × 10^11^)	40	4.16 × 10^11^ (1.16 × 10^12^)	5.04 × 10^11^ (1.75 × 10^12^)	38
*Lactobacillus* spp.	3.52 × 10^10^ (1.22 × 10^11^)	6.80 × 10^10^ (1.69 × 10^11^)	38	1.82 × 10^10^ (3.43 × 10^10^)	8.04 × 10^10^ * (1.82 × 10^11^)	37
*Clostridium* spp.	2.20 × 10^11^ (8.46 × 10^11^)	6.60 × 10^10^ (1.41 × 10^11^)	40	8.00 × 10^10^ (1.80 × 10^11^)	8.38 × 10^10^ (2.32 × 10^11^)	38

Asterisk denotes significant difference at *p* < 0.05, measured using one-way ANOVA. Data are presented as mean (standard deviation). Values represent number of bacteria (/g wet weight).

**Table 4:** Vital signs, body weight, and body mass index (BMI) of study participants assigned to the DW2009 and placebo groups.

Laboratory Profiles	Baseline			Follow-up		
	Placebo (*n* = 50)	DW2009 (*n* = 50)	*p*	Placebo (*n* = 47)	DW2009 (*n* = 45)	*p*
Systolic blood pressure (mmHg)	129 (13.3)	130 (13.5)	0.64	125 (13.4)	126 (12.8)	0.67
Diastolic blood pressure (mmHg)	78.3 (9.20)	81.4 (7.37)	0.06	76.2 (9.75)	77.4 (7.26)	0.52
Pulse rate (beats/min)	73.0 (10.1)	73.4 (11.1)	0.84	72.7 (7.58)	71.2 (8.64)	0.38
Body weight (kg)	60.3 (11.2)	63.2 (8.27)	0.15	60.0 (10.4)	62.6 (8.25)	0.19
Body mass index (kg/m^2^)	24.0 (3.41)	24.7 (3.16)	0.30	23.9 (3.16)	24.6 (3.31)	0.29

Data are presented as mean (standard deviation).

**Table 5:** Laboratory profiles of study participants assigned to the DW2009 and placebo groups.

Laboratory Profiles	Baseline			Follow-up		
	Placebo (*n* = 50)	DW2009 (*n* = 50)	*p*	Placebo (*n* = 47)	DW2009 (*n* = 45)	*p*
Complete blood cell count						
WBC (10^9^/L)	5.43 (1.16)	5.63 (1.19)	0.39	5.77 (1.51)	5.77 (1.45)	0.99
RBC (10^12^/L)	4.33 (0.33)	4.43 (0.33)	0.12	4.29 (0.32)	4.41 (0.30)	0.06
Hemoglobin (g/L)	134 (10.4)	137 (10.7)	0.23	133 (10.2)	136 (8.92)	0.11
Hematocrit (proportion of 1.0)	0.40 (0.03)	0.41 (0.03)	0.29	0.40 (0.03)	0.41 (0.03)	0.13
Platelet (10^9^/L)	239 (53.9)	246 (56.7)	0.55	241 (54.9)	248 (55.3)	0.57
Neutrophil (proportion of 1.0)	0.54 (0.08)	0.57 (0.09)	0.11	0.55 (0.09)	0.57 (0.08)	0.25
Blood chemistry						
Total Protein (g/L)	73.1 (4.16)	73.6 (3.76)	0.55	73.4 (3.75)	72.7 (3.62)	0.37
Albumin (g/L)	42.3 (1.88)	42.9 (2.14)	0.15	42.4 (1.75)	42.4 (1.84)	0.95
AST (µkat/L)	0.42 (0.11)	0.41 (0.11)	0.74	0.43 (0.13)	0.40 (0.10)	0.12
ALT (µkat/L)	0.33 (0.17)	0.35 (0.16)	0.47	0.34 (0.13)	0.32 (0.11)	0.63
ALP (µkat/L)	1.23 (0.32)	1.33 (0.50)	0.24	1.25 (0.31)	1.32(0.45)	0.43
Total Bilirubin (µmol/L)	14.4 (5.20)	15.1 (6.06)	0.58	13.1 (4.05)	14.4 (6.98)	0.26
Total Cholesterol (mmol/L)	5.23 (0.81)	5.02 (0.98)	0.26	5.08 (0.64)	4.97 (0.88)	0.51
Glucose (mmol/L)	5.32 (0.79)	5.30 (0.71)	0.92	5.36 (0.83)	5.31 (0.69)	0.79
Creatinine (µmol/L)	61.7 (13.5)	63.6 (13.3)	0.48	61.5 (13.3)	62.5 (14.3)	0.74
BUN (mmol/L)	5.50 (1.19)	5.56 (1.35)	0.80	5.73 (1.57)	5.89 (1.45)	0.63
Uric acid (µmol/L)	286 (78.1)	299 (71.0)	0.40	286 (75.0)	311 (69.8)	0.11

Data are presented as mean (standard deviation). Analyses for secondary efficacy measures were performed using a mixed-effects model repeated-measures analysis. Treatment group, visit, and treatment group-by-visit interaction were included as fixed effects, while the within-subjects factor was included as a random effect. WBC, white blood cell; RBC, red blood cell; AST, aspartate aminotransferase; ALT, alanine aminotransferase; ALP, alkaline phosphatase; BUN, blood urea nitrogen.
